# Randomised placebo-controlled clinical trial evaluating the impact of a new visual rehabilitation program on neuroadaptation in patients implanted with trifocal intraocular lenses

**DOI:** 10.1007/s10792-023-02809-9

**Published:** 2023-07-18

**Authors:** David P. Piñero, Miguel J. Maldonado-López, Ainhoa Molina-Martin, Noelia García-Sánchez, María L. Ramón, José L. Rincón, Alfredo Holgueras, Juan F. Arenillas, Álvaro Planchuelo-Gómez, Luis Leal-Vega, María Begoña Coco-Martín

**Affiliations:** 1 Group of Optics and Visual Perception, Department of Optics, Pharmacology and Anatomy, University of Alicante, Crta San Vicente del Raspeig S/N, 03016 San Vicente del Raspeig, Alicante Spain; 2 Department of Ophthalmology, Vithas Medimar International Hospital, Alicante, Spain; 3 Grupo de Cirugía Refractiva y Rehabilitación Visual, Instituto Universitario de Oftalmobiología Aplicada (IOBA), University of Valladolid, Valladolid, Spain; 4 Clínica Baviera, Valladolid, Spain; 5 Group of Applied Clinical Neurosciences and Advanced Data Analysis, Department of Medicine, Dermatology and Toxicology, University of Valladolid, Valladolid, Spain; 6 Stroke Unit and Stroke Program, Department of Neurology, University Clinical Hospital, University of Valladolid, Valladolid, Spain; 7 Image Processing Laboratory, University of Valladolid, Valladolid, Spain

**Keywords:** Trifocal intraocular lens, Neuroadaptation, Cataract surgery, Contrast sensitivity, Visual training, Multifocal IOL

## Abstract

**Purpose:**

To evaluate the efficacy of a new visual training program for improving the visual function in patients implanted with trifocal intraocular lenses (IOLs).

**Methods:**

Randomised placebo-controlled clinical trial enrolling 60 subjects (age, 47–75 years) undergoing cataract surgery with implantation of trifocal diffractive IOL. Home-based active visual training was prescribed immediately after surgery to all of them (20 sessions, 30 min): 31 subjects using a serious game based on Gabor patches (study group) and 29 using a placebo software (placebo group). Visual acuity, contrast sensitivity (CS), and perception of visual disturbances (QoV questionnaire) were evaluated before and after training. Likewise, in a small subgroup, resting-state functional magnetic resonance imaging (rs-fMRI) analysis was performed.

**Results:**

No significant differences were found between groups in compliance time (*p* = 0.70). After training, only significant improvements in monocular uncorrected intermediate visual acuity were found in the study group (*p* ≤ 0.01), although differences between groups did not reach statistical significance (*p* ≥ 0.11). Likewise, significantly better binocular far CS values were found in the study group for the spatial frequencies of 6 (*p* = 0.01) and 12 cpd (*p* = 0.03). More visual symptoms of the QoV questionnaire experienced a significant change in the level of bothersomeness in the study group. Rs-fMRI revealed the presence significant changes reflecting higher functional connectivity after the training with the serious game.

**Conclusions:**

A 3-week visual training program based on the use of Gabor patches after bilateral implantation of trifocal diffractive IOLs may be beneficial for optimising the visual function, with neural changes associated suggesting an acceleration of neuroadaptation.

*Trial registration* ClinicalTrials.gov, NCT04985097. Registered 02 August 2021, https://clinicaltrials.gov/(NCT04985097).

## Introduction

Crystalline lens extraction with implantation of a multifocal intraocular lens (IOL) has been shown to be a safe and efficacious option to provide a complete visual restoration in patients undergoing cataract surgery [1] and for presbyopia compensation in patients with clear lens [2]. However, visual disturbances, especially under scotopic conditions, are still one of the main concerns of this type of implants [3–5]. These include suboptimal visual acuity, decreased contrast sensitivity, and perception of photic phenomena, such as haloes or glare, that can lead to high levels of patient dissatisfaction [6] and in the most severe cases to the need for IOL explantation [7]. Excluding the presence of tear film instability, IOL decentration or posterior capsular opacification, these visual disturbances are mainly related to the neuroadaptation to the optical behaviour of the implant [8]. This process has been shown to be associated with increased activity in cortical areas involved in visual attention (frontoparietal circuits), learning and cognitive control (cingulate), and task goal-oriented behaviour in the early postoperative period (caudate), with normalisation six months after surgery [9, 10]. There is then a brain activity regularisation towards a non-effort pattern [9, 10]. In some cases, this neuroadaptation can fail leading to visual dissatisfaction and complaints in which other brain areas might be involved, such as corticostriatal loops that contribute to the interpretation of ambiguous visual scenes [10].

Some studies have shown that the visual function can be improved in patients implanted with multifocal IOLs by performing a well-oriented visual training program, suggesting the potential benefit of this approach for promoting or accelerating neuroadaptation [8, 11–13]. Kaymak et al. [12] reported the first results of a computer-based visual training experience in patients implanted with multifocal IOLs (diffractive bifocal and apodised diffractive), using only one eye for the training and serving the untrained fellow eye as a control. They reported an improvement in different visual parameters in spite of the limitations of the visual rehabilitation program used that was not gamified and used sinusoidal gratings without a smooth transition in their edges [12]. It should be considered that although the use of sinusoidal gratings with a neutral background can cause selective cortical responses for orientation and contrast [14], a stimulation of a specific channel is not achieved without an appropriate smoothing of the edges of the grating [15]. Recently, a new computerised game was developed to train the visual function of patients implanted with multifocal IOLs (OPTIcTRAIN®, Proconsi S.L., León, Spain), which is gamified and uses Gaussian-windowed sinewave gratings and exercises promoting the activation of frontoparietal circuits, cingulate, and caudate brain areas [11]. The preliminary results of the use of this game in patients implanted with trifocal IOLS are promising, providing significant improvements in distance and near contrast sensitivity [11].

Resting-state functional magnetic resonance imaging (rs-fMRI) is a safe and noninvasive technique that provides comprehensive and accurate data on brain anatomy, function and metabolism, making its incorporation into clinical studies highly recommended for assessing changes in functional connectivity produced by different therapies, including visual training [16]. This methodology has previously been applied in the field of vision for the general study of the various brain networks involved, such as the high-level visual network (HVN), the visuospatial network (VSN) or the primary visual network (PVN) [17], allowing comparison between healthy subjects and those with certain types of visual disorders. For example, Lu et al. [18] found a loss of functional correlation within the HVN and VSN by contrasting functional brain activity of 18 patients with anisometropic amblyopia against a sample of 18 age- and sex-matched healthy controls. In addition, rs-fMRI also makes it possible to visualise changes in the architecture of these networks in the same subject after a given treatment. This is the case of Halicka et al. [19], who evaluated the effects of 44 h of virtual reality-based active vision therapy over a period of 1.5 years on the visual cortex of an adult with anisometropic amblyopia. The use of rs-fMRI might be also useful to identify those changes occurring after the previously mentioned visual training in patients implanted with trifocal IOLs.

The aim of the current randomised placebo-controlled clinical trial was to evaluate the efficacy of this new visual training program for improving the visual function and minimising the perception of visual disturbances in a relevant sample of patients undergoing bilateral cataract surgery with the implantation of trifocal diffractive IOLs.

Furthermore, we aimed to assess functional and structural brain changes induced by therapy in these patients. On the one hand, a rs-fMRI substudy was performed on a small subsample of patients to confirm that the visual training program generated a differential impact on brain activity compared to the use of the placebo software. On the other hand, structural grey and white matter changes were assessed using morphometry parameters and diffusion MRI (dMRI) processing.

## Methods

### Design and settings

This was a blinded randomised placebo-controlled clinical trial. This clinical trial was registered in https://clinicaltrials.gov/(NCT04985097). The clinical trial protocol was approved by the ethics committee of the University of Alicante (UA-2019-02-20), as well as by the Medical Ethics Committee of the University Clinic Hospital of Valladolid (CASVE-NM-20-437). The trial was conducted in two Spanish centres between September 2020 and May 2021, the Department of Ophthalmology of the Vithas Medimar International Hospital (Alicante, Spain) and Clínica Baviera (Valladolid, España).

### Patients

Subjects who underwent uneventful bilateral cataract surgery with the implantation of trifocal diffractive IOLs, and that were willing to perform a visual training program in the immediate postoperative period, were included. Written informed consent was obtained from all participants before their enrolment in the study following the tenets of the Declaration of Helsinki.

Inclusion criteria were patients who undergone refractive lens exchange surgery for the correction of presbyopia with trifocal IOLs implantation at least 1 week before the evaluation visit for the trial, availability, and motivation to perform the assigned visual training, and availability to attend all follow-up visits. Exclusion criteria were patients implanted with other type of IOLs (monofocal, EDOF or refractive multifocal lenses) or having previous ocular surgeries, including laser corneal refractive surgery, intraoperative complications producing significant visual sequelae, presence of any active ocular disease or irregular cornea, illiteracy, and any type of psychological disorder.

Subjects were randomly assigned to use the visual training program (study group) or a placebo program after surgery (placebo group) using a random number sequence. The examiner was blinded to the type of training performed, training with the game using Gaussian-windowed sinewave gratings or training with the placebo software.

### Clinical protocol

Baseline visit was performed before the visual training, within 3 to 7 days after the surgery, including corneal topography, scotopic and photopic pupillometry, slit lamp biomicroscopy, manifest refraction, monocular and binocular uncorrected and corrected distance (6 m) (UDVA and CDVA), intermediate (1 m) (UIVA and CIVA) and near (40 cm) (UNVA and CNVA) visual acuity, monocular and binocular contrast sensitivity (CS) at far (4 m) using the CSV1000E test (VectorVision, Greenville, SC, USA), and binocular contrast sensitivity at near (40 cm) using the OPTIcTRAIN-CS® test. Visual acuity results were measured in decimal scale and were transformed to logarithmic scale for statistical purposes. CS results were measured in contrast threshold values and were transformed to logCS. Clinical data were complemented with the evaluation of patient subjective report of visual disturbances, such as halos or starbursts using the validated questionnaire developed by McAlinden et al. [20] QoV. The same procedures were repeated in the post-training visit, being the primary outcomes changes on visual acuity, CS and patient reported visual disturbances.

As described in a previous study of our research group [11], the OPTIcTRAIN-CS® test was specifically developed for this project and consisted of a near vision (40 cm) achromatic contrast sensitivity test based on best-PEST threshold strategy analysing the CS for the spatial frequencies of 0.5, 1.0, 1.5, 3.0, 4.5, and 6.0 cycles per degree (cpd) with a Gabor sinusoidal grating stimulus of 5 degrees. Characteristics of this test (measuring distance and conditions) and stimulus (frequencies, size and position) were the same than those used in the training for control group. CS measures were performed in a darkened room after an adaptation period of 5 min.

### Visual training

The visual training consisted of home-based active visual training by playing a serious game or a placebo software during 20 sessions of 30 min per day (that is 600 min in total). One or another program was installed in 20 hand-held devices (Samsung Tab A, Samsung, Suwon, South Korea) and were assigned randomly to the participants for training purposes, therefore determining the group. Twenty tablets were acquired and used for the training, and consequently, only twenty subjects could be simultaneously recruited for the trial. All screens were colorimetrically characterised using the Gain-offset-gamma method [21]. Subjects were trained in the use of the software, encouraging them to keep the viewing distance constant at 40 cm.

OPTIcTRAIN® software consisted of a serious game developed for the study purposes, based on a driving experience in which the subject must move right or left to avoid crashes with other vehicles and obstacles on the road. During the driving experience, Gabor stimulus popped on the road periodically, asking the driver the direction of the stimulus, and disappearing when an answer is done or following 5 s without an answer. Changes in the contrast of the stimuli were generated following the rules of the Best PEST psychophysical method [22]. A comprehensive description of this software is provided in our previous pilot study published [11]. The placebo software was a free-access game downloaded from PlayStore (Fun Kid Racing 3.53 for Android) based on a driving experience like the OPTIcTRAIN® software. The performance of both games from the subject point of view required the same implication from the driver, with the difference of the specific Gabor stimulation provided by OPTIcTRAIN® software.

Following the baseline visit, subjects were instructed in the use of the device and were appointed for the post-training visit 3 or 4 weeks later, to provide the time enough to develop the recommended 20 sessions. Furthermore, visual training was planned to be performed binocularly and without the use of optical correction.

### Compliance

Adherence to the visual training was evaluated by the evaluation of the level of compliance. The OPTIcTRAIN® software registers the game use every time the participant opens the application and plays the game, therefore automatically registering the real number of minutes dedicated to play with the game. In the case of the placebo software, a specific app was used to register its use by the participant. Participants were informed of this monitoring prior to participation and were not contacted during the training (to encourage their participation) to check their level of engagement with the software.

### MRI sub-study

A rs-fMRI scan was performed on a subgroup of five patients (four cases and one control) at the pre-intervention visit and at the post-intervention visit (after 30 days of therapy) to test the effects of visual training with the OPTIcTRAIN® software on the neural plasticity of the patients’ visual cortex. At both visits, T1-weighted and dMRI data were also acquired. Prior to MRI acquisition, patients signed a specific informed consent form after trained research staff verified that they met the necessary criteria for testing. All the MRI acquisitions took place on the Philips Achieva 3.0 T X-Series scanner at the Laboratory of Instrumental Techniques (LTI) of the Universidad de Valladolid.

For the purposes of this study, brain grey matter morphometry, white matter diffusion and functional connectivity analyses were performed. To quantify the variation in the different metrics between the two acquisitions (before and after the intervention), we used a coefficient of variation defined as (*M*_*t*0_ − *M*_*t*1_/*M*_*t*0_) × 100, where *M* is the metric under consideration, *t*_0_ corresponds to the first acquisition (pre-intervention visit) and *t*_1_ corresponds to the second acquisition (30-days follow-up).

For the analysis of brain grey matter, four parameters were selected: cortical curvature, cortical thickness, surface area and grey matter volume. These parameters were assessed in 34 bilateral cortical regions of interest (ROI). Moreover, the bilateral cerebellum and seven subcortical regions were exclusively analysed with the grey matter volume. The segmentation of the ROIs and the four morphometry parameters were obtained using the Freesurfer (v6.0.0) software standard pipeline for cortical parcellation. The aforementioned 84 regions were also employed to assess the functional connectivity.

For the analysis of brain white matter diffusion, three types of parameters were extracted:

- Parameters related to the diffusion tensor (classical): fractional anisotropy (FA), mean diffusivity (MD), axial diffusivity (AD) and radial diffusivity (RD).
- Parameters related to the diffusion propagator calculated with the AMURA (Apparent Measures Using Reduced Acquisitions) technique [23]: return-to-axis probability (RTAP), return-to-origin probability (RTOP) and return-to-plane probability (RTPP).
- Advanced parameters of the diffusion propagator using AMURA [24, 25]: propagator anisotropy (PA) and Q-space mean squared displacement (qMSD).

Specifically, the dMRI assessment was performed on 48 white matter regions, including some bilateral tracts such as the posterior corona radiata, posterior thalamic radiation (including optic radiation), and superior fronto-occipital fasciculus.

### Statistical analysis

Statistical analysis was performed by the software SPSS v. 22.0 for Windows (IBM, Armonk, NY, USA). The Kolmogorov–Smirnov test was used to determine whether the assessed variables followed a normal distribution, and therefore, if parametric or nonparametric statistical tests had to be applied. The significance of differences between study and placebo groups (two sample t-student or Mann–Whitney U test) and between pre and post-training visits (one sample t-student or Wilcoxon test) was analysed with the adequate tests depending on the normality of the compared variables according to the result from the Kolmogorov–Smirnov test. Likewise, the correlation between different evaluated variables was analysed, calculating the Pearson correlation coefficient for normal variables and the Spearman correlation coefficient for non-normally distributed variables. Differences in percentual variables between placebo and study groups were assessed with the Chi-square test, whereas differences between pre- and post-visual training visits were analysed with the McNemar test. Likewise, the raw QoV questionnaire data were Rasch-scaled onto an interval level scale. All statistical tests were 2-tailed, and *p*-values lower than 0.05 were considered statistically significant.

## Results

### Description of the sample

A total of 60 subjects were consecutively included, 29 in the placebo group (12 men and 17 women) and 31 in the study group (10 men and 21 women). The type of IOL implanted was Finevision POD F (PhysIOL, Liège, Belgium) in 43 of the patients, and RayOne (Rayner Intraocular Lenses Ltd., Worthing, UK) in 17 of the patients. Mean age was 65.73 ± 5.74 years in the placebo group and 63.45 ± 5.78 years in the study group, with no statistically significant differences between groups (*p* = 0.13).

In the placebo group, mean pupillary sizes under photopic and mesopic conditions in the right eyes were 2.24 ± 0.75 mm (range, 1.00 to 3.74) and 4.63 ± 0.58 mm (range, 3.08 to 5.67), respectively, whereas in the study group, these mean values were 2.23 ± 0.71 mm (range, 0.80 to 3.70) and 4.48 ± 0.58 mm (range, 2.85 to 5.75), with no statistically significant differences between groups (*p* = 0.93 and 0.33, respectively). In the left eyes, a similar finding was obtained, with mean photopic and scotopic pupil sizes of 2.31 ± 0.87 mm (range, 1.00 to 4.06) and 4.49 ± 0.47 mm (range, 3.47 to 5.26), respectively, in the placebo group, and mean values of 2.26 ± 0.92 mm (range, 0.90 to 4.36) and 4.43 ± 0.57 mm (range, 3.09 to 5.96) in the study group (*p* = 0.81 and 0.66, respectively).

No significant differences were found between placebo and study groups in terms of the IOL power implanted in the right eyes (placebo 22.8 ± 2.4 D vs. study 21.0 ± 4.1 D, *p* = 0.235). The same finding was obtained for the left eyes: placebo 22.8 ± 2.4 D vs. study 21.3 ± 3.9 D, *p* = 0.413). Anisometropia (difference in sphere between eyes of more than 1.50 D and in astigmatism of more than 1 D) was not observed in any case included.

Manifest refraction spherical equivalent before the visual training in the placebo group was − 0.10 ± 0.35 D (range, − 1.00 to + 1.00) in the right eyes, and 0.04 ± 0.41 D (range, − 0.75 to + 1.25) in the left eyes. In the study group, the spherical equivalent was − 0.13 ± 0.37 D (range, − 0.75 to + 0.50) in the right eyes, and − 0.04 ± 0.44 D (range, − 0.75 to + 1.25) in the left eyes. The difference between groups in the pre-training spherical equivalent of the right and left eyes did not reach statistical significance (*p* = 0.85 and 0.21, respectively). At the end of the visual training program, manifest refraction spherical equivalent in the placebo group was 0.00 ± 0.34 D (range, − 1.00 to + 0.75) and 0.01 ± 0.35 D (range, − 1.00 to + 1.00) in the right and left eyes, respectively, whereas these mean values in the study group were − 0.05 ± 0.30 D (range, − 0.75 to + 0.50) and 0.02 ± 0.32 D (range, − 0.50 to + 1.00), with no statistically significant differences between groups (*p* = 0.40 and 0.88, respectively).

All participants finished the training and attended all visits, although compliance with the training was not 100% in all cases. Mean compliance time was 477.67 ± 208.92 min (range, 30 to 720) and 518.16 ± 63.92 min (range, 390 to 600) in the placebo and study group, respectively. The differences between groups in this parameter were not statistically significant (*p* = 0.70).

### Uncorrected and corrected visual acuity

Uncorrected visual acuities for right eyes, left eyes, and binocular, before and after the training measured at far (6 m), intermediate (1 m) and near distances per group are summarised in Table 1. No statistically significant differences between groups were found for any visual acuity measured before (*p* ≥ 0.11 in all cases) and after the training (*p* ≥ 0.31 in all cases). Concerning the corrected visual acuities, a similar finding was obtained, with no significant differences between groups in any parameter measured before and after the training (*p* ≥ 0.29) (Table 2).

**Table 1:** Monocular and binocular uncorrected visual acuity (mean ± SD, and minimum–maximum) for distance, intermediate and near vision, before and after the visual training

			Uncorrected visual acuity		
			Group Placebo	Group Study	*p*
Pre-training	Distance	Right	0.05 ± 0.09 (− 0.08–0.35)	0.06 ± 0.06 (− 0.08–0.15)	0.11
		Left	0.07 ± 0.12 (− 0.08–0.46)	0.06 ± 0.08 (− 0.08–0.30)	0.98
		Binocular	0.01 ± 0.08 (− 0.10–0.35)	0.00 ± 0.06 (− 0.10–0.15)	0.68
	Intermediate	Right	0.19 ± 0.15 (0.00–0.50)	0.23 ± 0.16 (0.00–0.50)	0.36
		Left	0.20 ± 0.14 (0.00–0.40)	0.23 ± 0.15 (0.00–0.50)	0.42
		Binocular	0.14 ± 0.13 (− 0.08–0.30)	0.17 ± 0.14 (− 0.08–0.40)	0.47
	Near	Right	0.11 ± 0.10 (0.00–0.30)	0.12 ± 0.10 (0.00–0.30)	0.61
		Left	0.11 ± 0.12 (0.00–0.40)	0.11 ± 0.12 (0.00–0.52)	0.99
		Binocular	0.05 ± 0.08 (0.00–0.30)	0.06 ± 0.08 (0.00–0.22)	0.74
Post-training	Distance	Right	0.05 ± 0.11 (− 0.08–0.52)	0.05 ± 0.09 (− 0.08–0.35)	0.86
		Left	0.05 ± 0.11 (− 0.08–0.52)	0.05 ± 0.08 (− 0.08–0.30)	0.87
		Binocular	− 0.01 ± 0.08 (− 0.11–0.30)	− 0.00 ± 0.06 (− 0.10–0.15)	0.31
	Intermediate	Right	0.18 ± 0.13 (0.00–0.40)	0.16 ± 0.13 (0.00–0.50)	0.65
		Left	0.19 ± 0.13 (0.00–0.40)	0.18 ± 0.12 (0.00–0.40)	0.66
		Binocular	0.13 ± 0.14 (− 0.08–0.40)	0.15 ± 0.13 (− 0.08–0.40)	0.56
	Near	Right	0.10 ± 0.09 (0.00–0.30)	0.09 ± 0.10 (0.00–0.30)	0.67
		Left	0.10 ± 0.11 (0.00–0.40)	0.09 ± 0.13 (0.00–0.52)	0.49
		Binocular	0.05 ± 0.08 (0.00–0.30)	0.05 ± 0.09 (0.00–0.22)	0.81

**Table 2:** Monocular and binocular corrected visual acuity (mean ± SD, and minimum–maximum) for distance, intermediate and near vision, before and after the visual training

			Corrected visual acuity		
			Group Placebo	Group Study	*p*
Pre-training	Distance	Right	0.01 ± 0.05 (− 0.08–0.15)	0.02 ± 0.04 (− 0.08–0.10)	0.51
		Left	0.01 ± 0.05 (− 0.08–0.15)	0.01 ± 0.04 (− 0.08–0.10)	0.82
		Binocular	− 0.01 ± 0.04 (− 0.10–0.10)	− 0.01 ± 0.05 (− 0.10–0.06)	0.77
	Intermediate	Right	0.20 ± 0.16 (0.00–0.52)	0.23 ± 0.16 (0.00–0.50)	0.58
		Left	0.19 ± 0.14 (0.00–0.40)	0.24 ± 0.16 (0.00–0.54)	0.29
		Binocular	0.14 ± 0.13 (− 0.08–0.30)	0.17 ± 0.14 (− 0.08–0.40)	0.47
	Near	Right	0.10 ± 0.10 (0.00–0.30)	0.12 ± 0.09 (0.00–0.30)	0.49
		Left	0.08 ± 0.09 (0.00–0.30)	0.12 ± 0.12 (0.00–0.52)	0.38
		Binocular	0.05 ± 0.08 (0.00–0.30)	0.05 ± 0.08 (0.00–0.22)	0.57
Post-training	Distance	Right	0.01 ± 0.06 (− 0.08–0.22)	0.01 ± 0.05 (− 0.08–0.19)	0.90
		Left	0.01 ± 0.06 (− 0.08–0.22)	0.01 ± 0.05 (− 0.08–0.19)	0.64
		Binocular	− 0.02 ± 0.05 (− 0.10–0.15)	− 0.02 ± 0.05 (− 0.10–0.12)	0.52
	Intermediate	Right	0.18 ± 0.13 (0.00–0.40)	0.17 ± 0.13 (0.00–0.50)	0.82
		Left	0.19 ± 0.12 (0.00–0.40)	0.19 ± 0.12 (0.00–0.40)	0.96
		Binocular	0.13 ± 0.14 (− 0.08–0.30)	0.16 ± 0.13 (− 0.08–0.40)	0.36
	Near	Right	0.09 ± 0.09 (0.00–0.30)	0.08 ± 0.10 (0.00–0.30)	0.52
		Left	0.08 ± 0.09 (0.00–0.30)	0.09 ± 0.13 (0.00–0.52)	0.56
		Binocular	0.04 ± 0.07 (0.00–0.22)	0.05 ± 0.09 (0.00–0.22)	0.65

In the placebo group, changes after training in monocular (right: *p* = 0.61; left: *p* = 0.37) and binocular UDVA (*p* = 0.08) were not statistically significant. Similarly, changes with training in this group in monocular and binocular UIVA (right: *p* = 0.57; left: *p* = 0.77; binocular: *p* = 0.92) and UNVA (right: *p* = 0.76; left: *p* = 0.13; binocular: *p* = 0.50) did not reach either statistical significance.

In the study group, there were no significant changes after the visual training in the UDVA measured in the right eye (*p* = 0.08), left eye (*p* = 0.09) and binocularly (*p* = 0.17). However, a significant improvement was found in UIVA measured in right (*p* = 0.01) and left eyes (*p* < 0.01), but not binocularly (*p* = 0.61). As in the other group, no significant changes were found with training in monocular (right: *p* = 0.10; left: *p* = 0.16) and binocular UNVA (*p* = 0.63).

### Contrast sensitivity at far

**Fig. 1:**
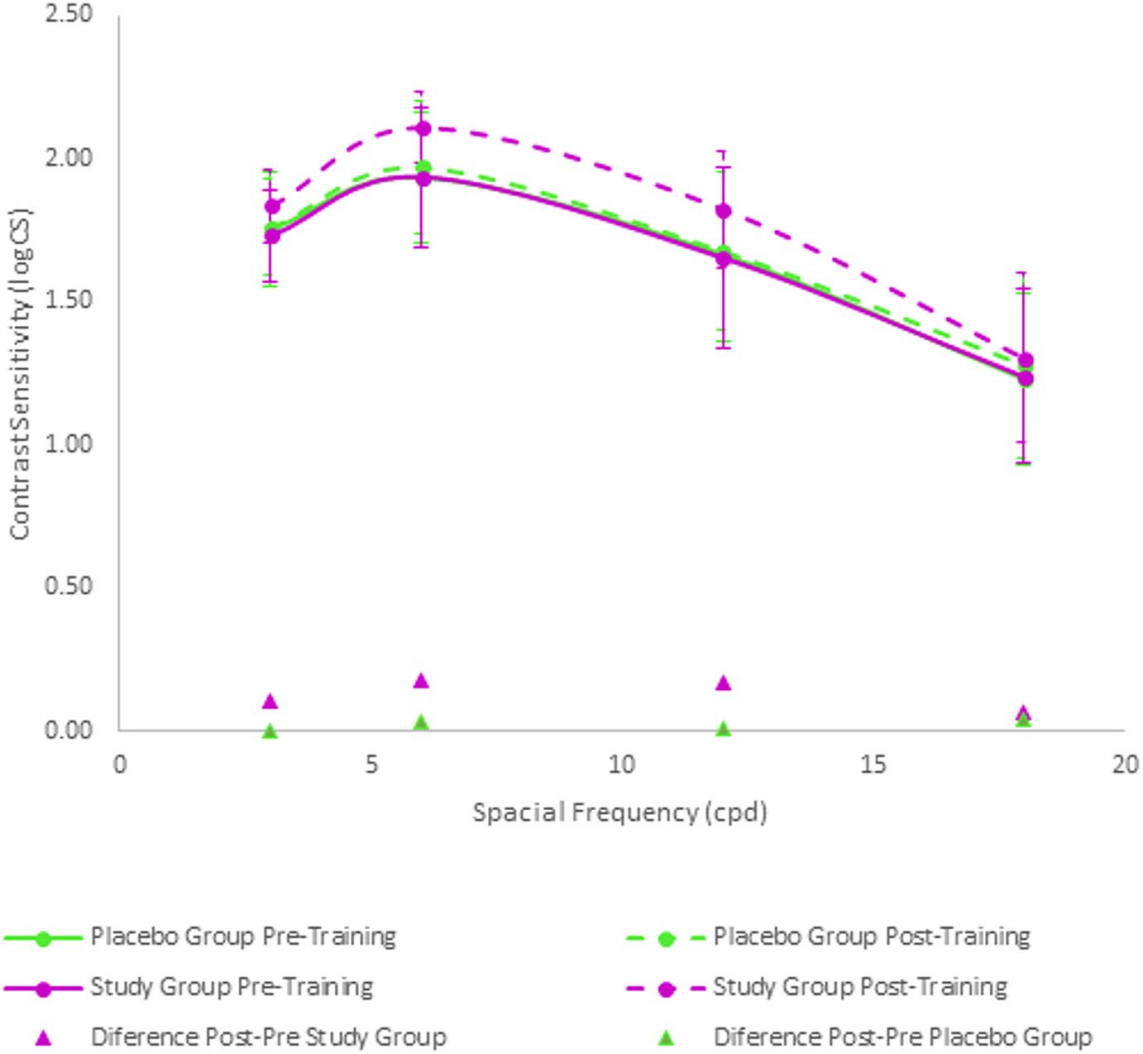
Binocular contrast sensitivity results at far measured with the CSV1000 test before and after the visual training. Δ represents the difference between the pre- and post-training data

Before the training, no significant differences between groups were found in monocular contrast sensitivity results (Table 3) (*p* ≥ 0.37). Likewise, no significant differences were present between the placebo and study group in binocular contrast sensitivity results before the training (Fig. 1) (*p* ≥ 0.46).

**Table 3:** Monocular contrast sensitivity results (mean ± SD, and minimum–maximum) measured at far for four spatial frequencies using the CSV1000 test before and after the visual training

			Monocular contrast sensitivity for distance vision		
			Group Placebo	Group Study	*p*
Pre-training	3 cpd	Right	1.68 ± 0.18 (1.34–2.08)	1.62 ± 0.21 (1.17–2.08)	0.37
		Left	1.59 ± 0.25 (1.00–1.93)	1.55 ± 0.36 (0.00–2.08)	0.64
	6 cpd	Right	1.86 ± 0.24 (1.38–2.14)	1.81 ± 0.44 (0.00–2.29)	0.96
		Left	1.86 ± 0.29 (1.21–2.29)	1.81 ± 0.41 (0.00–2.29)	0.72
	12 cpd	Right	1.51 ± 0.41 (0.00–1.99)	1.53 ± 0.48 (0.00–1.99)	0.46
		Left	1.42 ± 0.61 (0.00–1.99)	1.50 ± 0.40 (0.00–1.99)	0.85
	18 cpd	Right	1.13 ± 0.37 (0.47–2.08)	1.03 ± 0.41 (0.00–1.55)	0.67
		Left	1.03 ± 0.47 (0.00–1.55)	1.07 ± 0.43 (0.00–1.55)	0.84
Post-training	3 cpd	Right	1.76 ± 0.19 (1.17–2.08)	1.75 ± 0.14 (1.34–2.08)	0.51
		Left	1.61 ± 0.25 (1.00–1.93)	1.73 ± 0.17 (1.17–1.96)	0.60
	6 cpd	Right	1.90 ± 0.26 (1.38–2.29)	2.06 ± 0.17 (1.55–2.29)	0.01*
		Left	1.84 ± 0.28 (1.21–2.29)	1.99 ± 0.23 (1.38–2.29)	0.04.*
	12 cpd	Right	1.60 ± 0.33 (0.91–1.99)	1.63 ± 0.42 (0.00–1.99)	0.47
		Left	1.51 ± 0.48 (0.00–1.99)	1.66 ± 0.31 (0.91–1.99)	0.34
	18 cpd	Right	1.13 ± 0.35 (0.47–1.55)	1.21 ± 0.36 (0.00–1.55)	0.30
		Left	1.10 ± 0.41 (0.47–1.55)	1.17 ± 0.41 (0.00–1.55)	0.47

After the training, monocular contrast sensitivity results (Table 3) showed statistically significant differences in right and left eyes for the CS value measured for the spatial frequency of 6 (cpd) (right: *p* = 0.01; left: *p* = 0.04), with the best outcome in the study group. Binocularly, significantly better CS values were found in the study group for the spatial frequencies of 6 cpd (*p* = 0.01) and 12 cpd (*p* = 0.03) (Fig. 1).

Regarding longitudinal changes, no significant changes with training were found in the placebo group in most of monocular CS values (right *p* ≥ 0.09; left *p* ≥ 0.12) as well as in all binocular values (*p* ≥ 0.16). Only a statistically significant change was found in CS measured in the right eye for the spatial frequency of 3 cpd (*p* = 0.01). In contrast, in the study group, significant improvements with the visual training were found in monocular (right eye: *p* < 0.01 except for 12 cpd for which *p* = 0.12; left eye: *p* < 0.01 in all cases) and binocular CS (*p* < 0.01 in all cases).

### Contrast sensitivity at near

Before the training, CS results did not show statistically significant differences between groups for any of the spatial frequencies evaluated (*p* ≥ 0.33) (Table 4). After the training, results did not show either statistically significant differences between groups for any of the spatial frequencies analysed (*p* ≥ 0.14).

**Table 4:** Binocular contrast sensitivity results (mean ± SD, and minimum–maximum) at near measured with the OPTIcTRAIN® test before and after the visual training

		Binocular contrast sensitivity for near vision		
		Group placebo	Group study	*p*
Pre-training	0.5 cpd	1.02 ± 0.60 (0.35–2.00)	0.98 ± 0.55 (0.33–2.00)	0.87
	1.0 cpd	1.57 ± 0.57 (0.38–2.00)	1.45 ± 0.55 (0.60–2.00)	0.33
	1.5 cpd	1.61 ± 0.51 (0.69–2.00)	1.51 ± 0.52 (0.71–2.00)	0.47
	3.0 cpd	1.19 ± 0.53 (0.38–2.00)	1.22 ± 0.62 (0.15–2.00)	0.99
	4.5 cpd	1.00 ± 0.73 (0.18–2.00)	0.81 ± 0.60 (0.05–2.00)	0.56
	6.0 cpd	0.62 ± 0.48 (0.05–2.00)	0.69 ± 0.53 (0.15–2.00)	0.75
Post-training	0.5 cpd	1.08 ± 0.52 (0.26–2.00)	1.19 ± 0.61 (0.32–2.00)	0.50
	1.0 cpd	1.51 ± 0.58 (0.38–2.00)	1.49 ± 0.54 (0.60–2.00)	0.86
	1.5 cpd	1.51 ± 0.58 (0.22–2.00)	1.72 ± 0.45 (1.02–2.00)	0.14
	3.0 cpd	1.24 ± 0.56 (0.38–2.00)	1.31 ± 0.56 (0.38–2.00)	0.73
	4.5 cpd	0.87 ± 0.57 (0.28–2.00)	1.03 ± 0.65 (0.33–2.00)	0.31
	6.0 cpd	0.55 ± 0.31 (0.06–1.02)	0.55 ± 0.39 (0.14–2.00)	0.82

Concerning the longitudinal analysis in each group, no statistically significant changes were detected with training in any CS value measured (placebo group: 0.5 cpd, *p* = 0.70, 1 cpd *p* = 0.51, 1.5 cpd *p* = 0.45, 3 cpd *p* = 0.78, 4.5 cpd *p* = 0.51, 6 cpd *p* = 0.51; study group: 0.5 cpd *p* = 0.15, 1 cpd *p* = 0.42, 1.5 cpd *p* = 0.10, 3 cpd *p* = 0.72, 4.5 cpd *p* = 0.14, 6 cpd *p* = 0.15).

### Visual quality outcomes

Visual quality outcomes evaluated by means of the QoV questionnaire before and after the training are reported in Tables 5 and 6. As can be seen in these tables, there were no statistically significant differences between groups in the evaluation of the frequency, severity and bothersomeness of the different visual disturbances evaluated before (*p* ≥ 0.11) or after the training (*p* ≥ 0.10).

**Table 5:** Quality of Vision questionnaire results obtained before the training in the placebo and study groups. Results were represented in percentage (and number of subjects) for frequency, severity and bothersomeness of the different disturbances evaluated

		Never/not at all		Occasionally/mild/a little		Quite often/moderate		Very often/Severe		*p* value
		Placebo	Study	Placebo	Study	Placebo	Study	Placebo	Study	
Glare	Frequency	68% (19)	71% (22)	14% (4)	10% (3)	11% (3)	6.5% (2)	7% (2)	13% (4)	0.78
	Severity	68% (19)	71% (22)	0% (0)	6.5% (2)	21% (6)	19% (6)	11% (3)	3% (1)	0.38
	Bothersome	68% (19)	77% (24)	14% (4)	6.5% (2)	11% (3)	13% (4)	7% (2)	3% (1)	0.66
Halos	Frequency	14% (4)	13% (4)	14% (4)	26% (8)	21% (6)	10% (3)	50% (14)	52% (16)	0.51
	Severity	18% (5)	13% (4)	18% (5)	13% (4)	29% (8)	55% (17)	36% (10)	19% (6)	0.23
	Bothersome	29% (8)	32% (10)	25% (7)	39% (12)	36% (10)	23% (7)	11% (3)	6% (2)	0.55
Starburst	Frequency	64% (18)	68% (21)	18% (5)	23% (7)	3.6% (1)	6%(2)	14% (4)	3% (1)	0.47
	Severity	64% (18)	77% (24)	14% (4)	10% (3)	18% (5)	10% (3)	4% (1)	3% (1)	0.72
	Bothersome	64% (18)	81% (25)	21% (6)	16% (5)	14% (4)	0% (0)	0% (0)	3% (1)	0.11
Haze	Frequency	71% (20)	77% (24)	21% (6)	13% (10)	4% (1)	3% (1)	4% (1)	6% (2)	0.81
	Severity	71% (20)	81% (25)	4% (1)	10% (3)	21% (6)	6% (2)	4% (1)	3% (1)	0.33
	Bothersome	71% (20)	84% (26)	7% (2)	3% (1)	18% (5)	10% (3)	4% (1)	3% (1)	0.69
Blur	Frequency	71% (20)	58% (18)	21% (6)	19% (6)	0% (0)	16% (5)	7% (2)	6% (2)	0.17
	Severity	71% (20)	71% (22)	7% (2)	10% (3)	18% (5)	6% (2)	4% (1)	13% (4)	0.65
	Bothersome	71% (20)	71% (22)	11% (3)	10% (3)	14% (4)	6% (2)	4% (1)	13% (4)	0.49
Distortion	Frequency	82% (23)	87% (27)	14% (4)	3% (1)	4% (1)	6% (2)	0% (0)	3% (1)	0.35
	Severity	82% (23)	90% (28)	11% (3)	0% (0)	7% (2)	10% (3)	0% (0)	0% (0)	0.17
	Bothersome	82% (23)	93% (29)	11% (3)	0% (0)	7% (2)	6% (2)	0% (0)	0% (0)	0.17
Double vision	Frequency	93% (26)	90% (28)	0% (0)	6% (2)	7% (2)	3% (1)	0% (0)	0% (0)	0.32
	Severity	93% (26)	90% (28)	0% (0)	10% (3)	4% (1)	0% (0)	4% (1)	0% (0)	0.18
	Bothersome	93% (26)	93% (29)	0% (0)	3% (1)	7% (2)	3% (1)	0% (0)	0% (0)	0.51
Fluctuation	Frequency	71% (20)	77% (24)	21% (6)	16% (5)	4% (1)	3% (1)	4% (1)	3% (1)	0.96
	Severity	71% (20)	81% (25)	21% (6)	13% (4)	4% (1)	3% (1)	4% (1)	3% (1)	0.85
	Bothersome	71% (20)	84% (26)	21% (6)	10% (3)	4% (1)	3% (1)	4% (1)	3% (1)	0.65
Focusing difficulties	Frequency	68% (19)	68% (21)	21% (6)	16% (5)	7% (2)	10% (3)	4% (1)	6% (2)	0.90
	Severity	71% (20)	71% (22)	14% (4)	19% (6)	11% (3)	10% (3)	4% (1)	0% (0)	0.72
	Bothersome	71% (20)	71% (22)	21% (6)	19% (6)	4% (1)	6% (2)	4% (1)	3% (1)	0.96
Depth Perception difficulty	Frequency	82% (23)	93% (29)	11% (3)	3% (1)	7% (2)	3% (1)	0% (0)	0% (0)	0.39
	Severity	82% (23)	93% (29)	11% (3)	3% (1)	4% (1)	0% (0)	4% (1)	3% (1)	0.47
	Bothersome	82% (23)	93% (29)	11% (3)	3% (1)	7% (2)	3% (1)	0% (0)	0% (0)	0.39

**Table 6:** Quality of Vision questionnaire results obtained after the training in the placebo and study groups. Results were represented in percentage (and number of subjects) for frequency, severity and bothersomeness of the different disturbances evaluated

		Never/not at all		Occasionally/mild/a little		Quite often/moderate		Very often/severe		*P* value
		Placebo	Study	Placebo	Study	Placebo	Study	Placebo	Study	
Glare	Frequency	71% (20)	68% (21)	18% (5)	26% (8)	4% (1)	3% (1)	7% (2)	3% (1)	0.82
	Severity	71% (20)	71% (22)	14% (4)	13% (4)	11% (3)	16% (5)	4% (1)	0% (0)	0.69
	Bothersome	75% (21)	71% (22)	14% (4)	19% (6)	7% (2)	10% (3)	4% (1)	0% (0)	0.69
Halos	Frequency	4% (1)	6% (2)	18% (5)	23% (7)	29% (8)	19% (6)	50% (14)	52% (16)	0.82
	Severity	7% (2)	10% (3)	29% (8)	13% (4)	32% (9)	45% (14)	32% (9)	32% (10)	0.47
	Bothersome	25% (7)	26% (8)	36% (10)	42% (13)	25% (7)	29% (8)	14% (4)	3% (1)	0.50
Starburst	Frequency	82% (23)	77% (24)	14% (4)	3% (1)	4% (1)	6% (2)	0% (0)	13% (4)	0.11
	Severity	82% (23)	81% (25)	18% (5)	6% (2)	0% (0)	6% (2)	0% (0)	6% (2)	0.16
	Bothersome	89% (25)	81% (25)	11% (3)	16% (5)	0% (0)	3% (1)	0% (0)	0% (0)	0.51
Haze	Frequency	89% (25)	87% (27)	7% (2)	13% (4)	4% (1)	0% (0)	0% (0)	0% (0)	0.45
	Severity	89% (25)	87% (27)	0% (0)	6% (2)	7% (2)	6% (2)	4% (1)	0% (0)	0.40
	Bothersome	89% (25)	87% (27)	7% (2)	10% (3)	4% (1)	3% (1)	0% (0)	0% (0)	0.94
Blur	Frequency	68% (19)	74% (23)	25% (7)	16% (5)	7% (2)	3% (1)	0% (0)	6% (2)	0.41
	Severity	68% (19)	74% (23)	21% (6)	19% (6)	11% (3)	6% (2)	0% (0)	0% (0)	0.81
	Bothersome	71% (20)	74% (23)	14% (4)	23% (7)	14% (4)	3% (1)	0% (0)	0% (0)	0.26
Distortion	Frequency	96% (27)	84% (26)	4% (1)	13% (4)	0% (0)	3% (1)	0% (0)	0% (0)	0.26
	Severity	96% (27)	84% (26)	4% (1)	16% (5)	0% (0)	0% (0)	0% (0)	0% (0)	0.11
	Bothersome	96% (27)	87% (27)	4% (1)	13% (4)	0% (0)	0% (0)	0% (0)	0% (0)	0.20
Double vision	Frequency	96% (27)	90% (28)	4% (1)	6% (2)	0% (0)	3% (1)	0% (0)	0% (0)	0.55
	Severity	96% (27)	90% (28)	4% (1)	6% (2)	0% (0)	3% (1)	0% (0)	0% (0)	0.55
	Bothersome	96% (27)	90% (28)	4% (1)	6% (2)	0% (0)	3% (1)	0% (0)	0% (0)	0.55
Fluctuation	Frequency	75% (21)	90% (28)	25% (7)	6% (2)	0% (0)	3% (1)	0% (0)	0% (0)	0.10
	Severity	75% (21)	90% (28)	25% (7)	6% (2)	0% (0)	3% (1)	0% (0)	0% (0)	0.10
	Bothersome	75% (21)	90% (28)	25% (7)	6% (2)	0% (0)	3% (1)	0% (0)	0% (0)	0.10
Focusing difficulties	Frequency	79% (22)	71% (22)	14% (4)	23% (7)	4% (1)	6% (2)	4% (1)	0% (0)	0.57
	Severity	79% (22)	74% (23)	14% (4)	19% (6)	7% (2)	6% (2)	0% (0)	0% (0)	0.87
	Bothersome	86% (24)	77% (24)	7% (2)	23% (7)	7% (2)	0% (0)	0% (0)	0% (0)	0.10
Depth Perception difficulty	Frequency	93% (26)	87% (27)	4% (1)	13% (4)	4% (1)	0% (0)	0% (0)	0% (0)	0.26
	Severity	93% (26)	90% (28)	4% (1)	10% (3)	4% (1)	0% (0)	0% (0)	0% (0)	0.38
	Bothersome	93% (26)	93% (29)	4% (1)	6% (2)	4% (1)	0% (0)	0% (0)	0% (0)	0.51

In the placebo group, statistically significant changes were found with training in the frequency of blur (*p* < 0.01) and depth perception difficulty (*p* < 0.01), the severity of blur (*p* < 0.01), double vision (*p* < 0.01) and depth perception difficulty (*p* = 0.01), and the level of bothersomeness of blur (*p* < 0.01) and depth perception difficulty (*p* < 0.01). In the study group, a significant change with training was found in the frequency of distortion (*p* < 0.01) and vision fluctuation (*p* = 0.02), the severity of blur (*p* = 0.02), distortion (*p* < 0.01), double vision (*p* < 0.01), and vision fluctuation (*p* = 0.02), and the level of bothersomeness of glare (*p* < 0.01), starbursts (*p* < 0.01), distortion (*p* < 0.01), and vision fluctuation (*p* = 0.01).

**Fig. 2:**
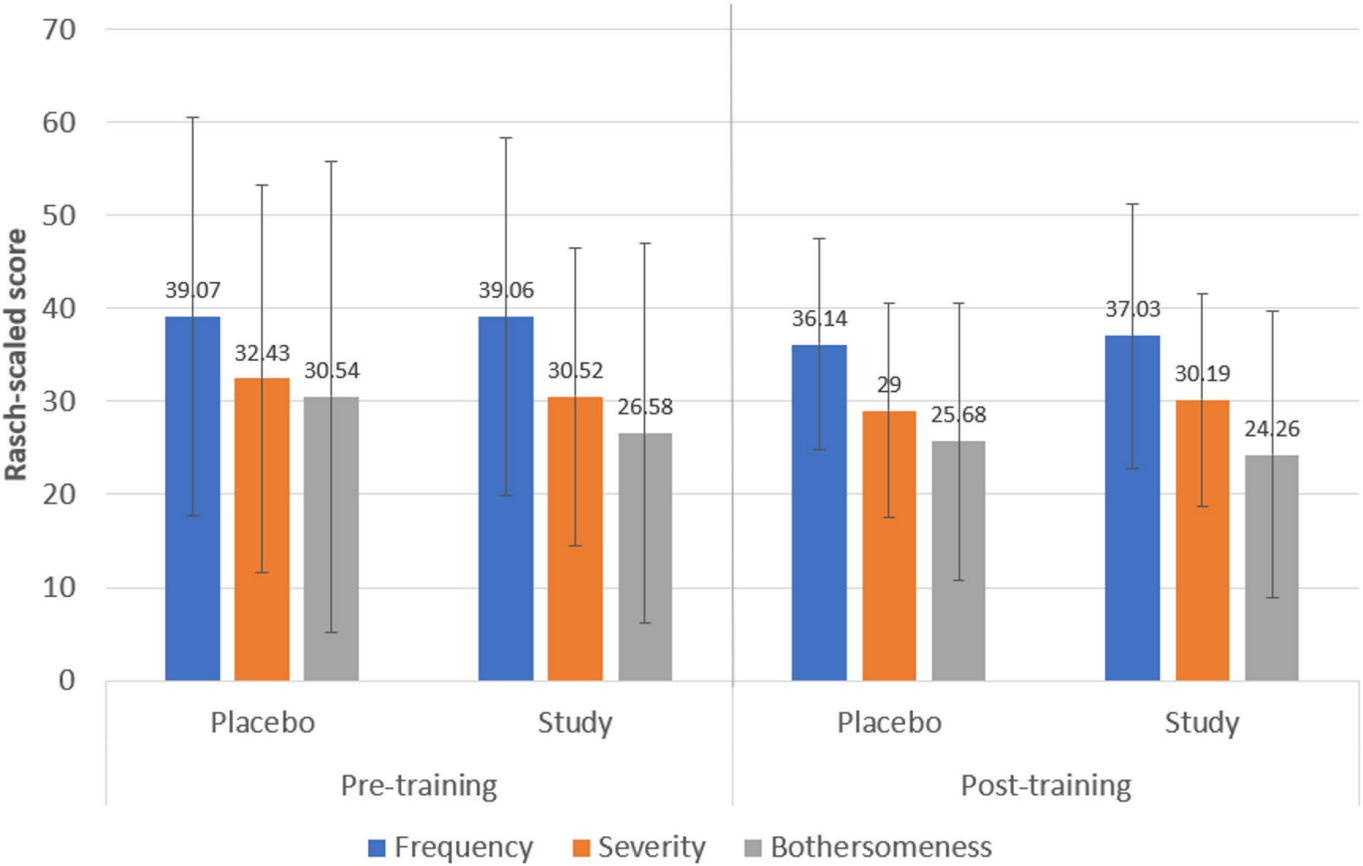
Rasch-scale scores of frequency, severity and bothersomeness of quality of vision disturbances before and after training

Once the raw questionnaire data were Rasch-scaled onto an interval level scale, the mean values are displayed in Fig. 2. No significant differences were found between groups before training in any of the Rasch-scaled scores obtained for frequency (*p* = 0.891), severity (*p* = 0.831), and bothersomeness (*p* = 0.591) of visual quality disturbances. After training, differences between groups did not reach statistical significance (frequency *p* = 0.951, severity *p* = 0.926, bothersomeness *p* = 0.558), although in both groups there was a trend to a reduction in these scores (*p* ≥ 0.191).

### MRI substudy: grey matter morphometry analysis

Regarding the cortical curvature, statistically significant longitudinal changes with training in the four patients from the study group were observed in the following areas (all in the right hemisphere): decrease in postcentral (*p* = 0.016), decrease in precuneus (*p* = 0.034), decrease in superior parietal (*p* = 0.025), and increase in pars triangularis (*p* = 0.005). Statistically significant changes of surface area were observed in the following ROIs: decrease in right supramarginal gyrus (*p* = 0.024), increase in left parahippocampal gyrus (*p* = 0.025), decrease in left temporal inferior gyrus (*p* = 0.012), and decrease in right banks of the superior temporal sulcus (*p* = 0.024). Regarding cortical thickness and grey matter volume, no statistically significant changes were observed (*p* > 0.05).

### MRI substudy: white matter diffusion analysis

In terms of AD, statistically significant increases were observed in the four patients from the study group in the right medial lemniscus (*p* = 0.036) and in the left retrolenticular part of internal capsule (*p* = 0.022). Furthermore, significant decreases were observed in FA in the right cingulum (hippocampus) (*p* = 0.015) and in the left uncinate fasciculus (*p* = 0.039). Likewise, in terms of MD, statistically significant changes were observed in the following areas: increase in left sagittal stratum (*p* = 0.028), increase in right uncinate fasciculus (*p* < 0.001), increase in left uncinate fasciculus (*p* = 0.034), and increase in left retrolenticular part of internal capsule (*p* < 0.001). In terms of RD, statistically significant increases were observed in the right cingulum (hippocampus) (*p* = 0.030), left cingulum (hippocampus) (*p* = 0.019), right uncinate fasciculus (*p* = 0.018), left uncinate fasciculus (*p* = 0.012), left superior longitudinal fasciculus (*p* = 0.048) and left retrolenticular part of the internal capsule (*p* = 0.015).

As for the values related to the diffusion propagator using the AMURA technique, significant changes were detected in RTAP, RTOP, RTPP, PA and qMSD. In terms of RTAP, statistically significant decreases were observed in the left uncinate fasciculus (*p* = 0.022) and in the left retrolenticular part of internal capsule (*p* = 0.022). Likewise, statistically significant decreases were seen in RTOP in the left uncinate fasciculus (*p* = 0.014), right uncinate fasciculus (*p* = 0.034), and left retrolenticular part of internal capsule (*p* = 0.005). Concerning RTPP, a statistically significant increase was observed in the right posterior thalamic radiation (*p* = 0.021) and a significant decrease in the left anterior limb of internal capsule (*p* = 0.028).

As for the values related to the advanced parameters of diffusion propagation, statistically significant decreases in terms of PA were observed in the left retrolenticular part of the internal capsule (*p* = 0.020), right cingulum (hippocampus) (*p* = 0.030), left cingulum (hippocampus) (*p* = 0.005) and left uncinate fasciculus (*p* = 0.045). In turn, statistically significant decreases in terms of qMSD were observed in the left retrolenticular part of the internal capsule (*p* = 0.022), the right external capsule (*p* = 0.031), the right uncinate fasciculus (*p* = 0.025) and the left uncinate fasciculus (*p* = 0.043).

### MRI substudy: functional connectivity analysis

**Fig. 3:**
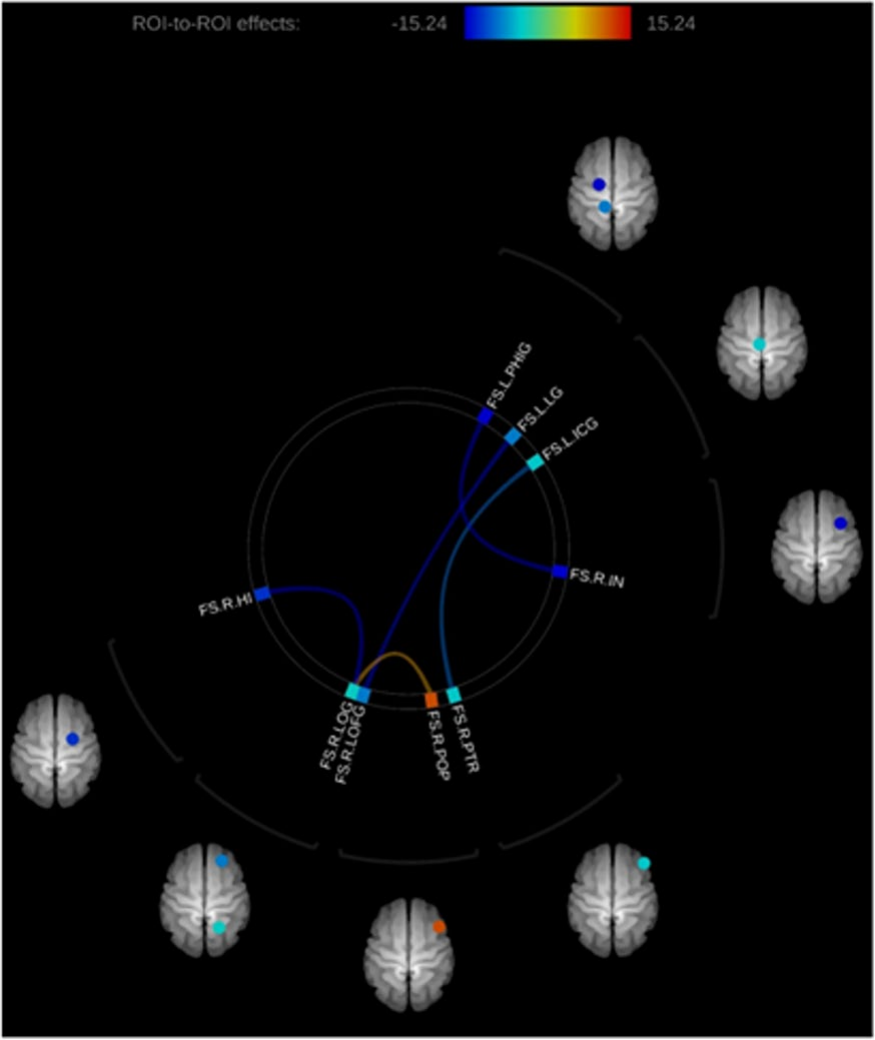
Connections with significant differences after therapy. Blue connections mean lower functional connectivity before than after the intervention, and orange connections mean higher functional connectivity before than after the intervention. *FS* FreeSurfer; *L-PHIG* left parahippocampal gyrus; *L-LG* left lingual gyrus; *L-ICG* left isthmus cingulate gyrus; *R-IN* right insula; *R-PTR* right pars triangularis; *R-POP* right pars opercularis; *R-LOFG* right lateral orbitofrontal gyrus; *R-LOG* right lateral occipital gyrus; *R-HI* right hippocampus

Most statistically significant changes reflected higher functional connectivity after the intervention. In relation to grey matter regions associated with visual processing, significant lower functional connectivity before the intervention was found in the connection between the right lateral occipital gyrus and the right hippocampus, and between the left lingual gyrus and left lateral orbital frontal gyrus. In contrast, higher functional connectivity before the intervention was appreciated in the connection between the right lateral occipital gyrus and the right pars opercularis. Moreover, other connections with significant higher connectivity after the intervention involved the left parahippocampal gyrus, left isthmus cingulate gyrus, right insula, and right pars triangularis. These results are shown with higher detail in Fig. 3.

## Discussion

The current placebo-controlled clinical trial evaluates the potential benefit of a Gabor gratings-based visual training program after trifocal diffractive IOL implantation. In the sample evaluated, no significant differences between placebo and study groups were found in uncorrected and distance-corrected visual outcomes, with outcomes comparable to those obtained by previous authors with the two trifocal IOLs used in the study [11, 26–29]. However, a small but statistically significant improvement was found in the study group in monocular UIVA for right and left eyes. It should be noted here that the far and near visual outcomes obtained with the two types of trifocal IOLs included in the current series were already good in the initial postoperative period, with only some level of visual limitation at intermediate distance. Therefore, the visual training program evaluated seems to improve the intermediate visual function. There are previous experiences demonstrating that the visual training based on the visualisation of Gabor gratings with different contrasts can induce improvements in visual acuity [30, 31]. Tan and Fong [29] found a significant improvement in uncorrected visual acuity and contrast sensitivity in low myopic eyes performing a Gabor gratings-based visual training. In our trial, as the refractive predictability with the IOLs implanted was very high, the residual refraction was minimal, not affecting distance visual acuity and not being necessary the adaptation to a specific level of residual refraction.

Significant differences between groups were found in the current sample after the training in distance CS outcomes, with better results after the training in the study group for the monocular and binocular values obtained for the spatial frequency of 6 cpd as well as for the binocular values corresponding to the spatial frequency of 12 cpd. Furthermore, significant improvements were only found in the study groups for almost all CS data obtained. This confirms the positive impact of the Gabor gratings-based training on CS and consequently on the quality of vision achievable at distance with the trifocal IOLs evaluated. This is consistent with previous experiences of visual training using sinusoidal gratings in eyes implanted with multifocal IOLs [11–13]. Our research group recently published a pilot experience with the same visual training program in eyes implanted with trifocal diffractive IOLs, also obtaining significantly better distance CS for the spatial frequencies of 6 (*p* = 0.02) and 12 cpd (*p* = 0.01) compared to a placebo group [11]. Kaymak et al. [12] trained one eye of 16 patients implanted with apodised diffractive bifocal IOLs using a computerised system based on the concept of the perceptual learning of discrimination line orientations, with six training sessions of a mean duration of 30 ± 5 min over 2 weeks. The untrained fellow eye was used as the control. These authors reported significant improvements after the training in orientation visual acuity and the area under the CS curve under photopic, mesopic, and mesopic with glare conditions compared to control eyes [12]. Concerning changes in near CS, they did not achieve statistical significance in the current trial in any of the two groups, although there was a trend to improve in the study group, especially for the spatial frequency of 1.5 cpd. In a previous pilot study for evaluating the potential benefit of the same visual training program, our research group found significantly better values of near CS compared to a placebo group for the spatial frequency of 1.5 cpd [11]. More studies are needed to confirm the potential changes in near CS with the Gabor gratings-based visual training program evaluated, even using other measurement methodologies to confirm the outcome.

Patient’s reported quality of vision by means of a validated questionnaire was also evaluated, with no significant differences between study and placebo groups in the whole Rasch-scaled scores for the frequency, severity and bothersomeness of visual disturbances, although a non-significant trend to a decrease in such scores was found after training in both groups. Likewise, no significant differences between groups were found either in the distribution of the frequency, severity and bothersomeness scores for a great variety of visual symptoms after the training, including halos, glare, and starbursts. However, significant changes between pre- and post-training visits were found in both groups in the distribution of frequency, severity and bothersomeness of some symptoms. Specifically, in the placebo group, a significant change of the distribution of blur and depth perception difficulty was found after training, with a trend to lower scores, representing lower frequency of these symptoms as well as less level of severity and bothersomeness. In the study group, a significant change towards lower scores of frequency, severity and bothersomeness was detected for distortion and vision fluctuation. Likewise, a significant change towards lower levels of bothersomeness was also found after training for glare and starbursts. In both groups, a significant change in the severity of double vision was found, being significantly lower after training. Therefore, some changes in visual symptoms, with a clear trend to reduction over time, were found in both groups which reveals that the process of neuroadaptation is initiated independently of the training. The only difference is the significant change in the study group of the level of bothersomeness of more visual symptoms, including glare and starbursts, that may be related to this potential process of acceleration of neuroadaptation promoted with the Gabor gratings-based visual training. This should be confirmed in future studies including different methodologies to evaluate the patient’s visual quality, such as other validated questionnaires, distorsiometry or halometry. To this date, this is the first study investigating the impact on patient reported visual quality of visual training in subjects implanted with multifocal IOLs.

**Fig. 4:**
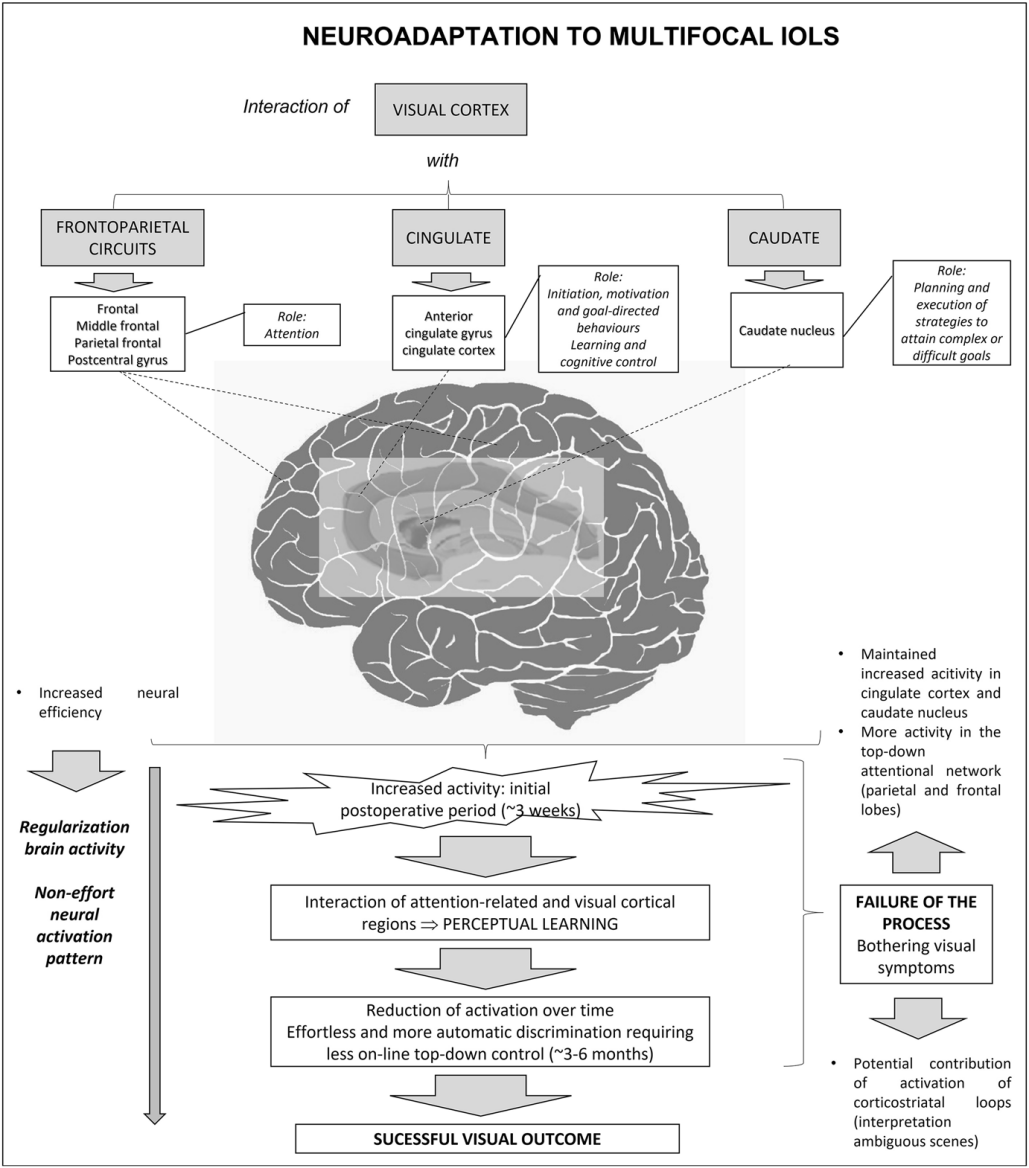
Summary of the processes and brain areas involved in neuroadaptation to multifocal IOLs according to Rosa et al. [9, 10]

Finally, in a small subgroup of patients, the neural impact of the visual training was investigated using MRI data. A total of 4 eyes trained with the Gabor gratings-based software were evaluated with rs-fMRI and structural MRI (dMRI and T1-weighted-based morphometry), as well as one patient trained with the placebo. Some significant changes were found despite the small sample. Specifically, a significant reduction in the cortical curvature parameter, which is consistent with the change to a most optimal status, was observed in the following areas of the right hemisphere: postcentral, precuneus, and superior parietal. In contrast, the changes observed for the surface area followed an inconsistent pattern, with statistically significant increase and decrease after the intervention. Regarding the analysis of brain white matter diffusion, it should be considered that lower FA is usually related to pathological or worse state, whereas for the rest of the parameters (AD, MD, RD), the tendency is usually the opposite. In the four eyes from the study group, significant increases were observed in AD in the right medial lemniscus and in the left retrolenticular part of internal capsule, as well as significant decreases in FA in the right cingulum (hippocampus) and in the left uncinate fasciculus. A significant increase was also observed in RD in the right cingulum as well as a significant increase in MD and RD in the left uncinate fasciculus. The left retrolenticular part of the internal capsule also experiences a significant increase in MD and RD. It should be considered that changes in the cingulate area have been shown to be related to the process of neuroadaptation to multifocality, with a regularisation of activity towards a non-effort pattern (Fig. 4) [9, 10]. Besides these changes, significant increases in MD were found in left sagittal stratum, and right uncinate fasciculus as well as significant increases in the left cingulum, right uncinate fasciculus, and left superior longitudinal fasciculus. All these changes are consistent with this trend to a non-effort pattern, which is consistent with expertise acquisition and performance improvement.

In terms of the diffusion propagator using the AMURA technique, significant decreases were also found in RTAP, RTOP, PA and qMSD in the left uncinate fasciculus and in the left retrolenticular part of internal capsule. Other significant decreases found were the following: RTOP and qMSD in right uncinate fasciculus, RTPP in the left anterior limb of internal capsule, PA in right cingulum and left cingulum (hippocampus), and qMSD in right external capsule. As happened with the diffusion tensor analysis, a change to an enhanced status was found in the cingulate area, which has an important role in attention, goal-directed behaviours and error monitoring (Fig. 4), being consistent with the results of previous studies confirming the neural changes required for a successful neuroadaptation [9, 10]. It should be considered that this process is initiated with an increased activity in the top-down attentional network, cingulate cortex and caudate nucleus in the initial 3 weeks after surgery that evolves to a cortical regularisation of activity towards a non-effort pattern. Our results seem to indicate some level of acceleration of this process, with the non-effort pattern already initiated during this 3-week visual training period. This should be confirmed in future series including the neural activity evaluation in larger series. Concerning the functional connectivity analysis, except for the connection between the pars opercularis and the right lateral occipital gyrus, functional connectivity increased after the intervention.

This clinical trial had some limitations that should be acknowledged. First, the follow-up is short and future studies must be conducted to evaluate these outcomes in the long term. Only Kaymak et al. [12] have investigated to this date the maintenance of the result of a visual training program based on the concept of perceptual learning of discrimination line orientations over a 6-month period. The inclusion of two different types of multifocal IOLs may be considered another limitation, but it should be considered that the two types of trifocal IOLs have diffractive profile and have demonstrated to provide very similar visual outcomes [25]. Ribeiro and Ferreira [25] evaluated the clinical outcomes of the two trifocal IOLs used in the current study and found that there were no significant differences between them in terms of distance, intermediate and near visual outcomes. Likewise, our research group demonstrated in a previous pilot study that there were no significant differences in the results of the visual training with the Gabor gratings-based software evaluated between patients implanted with the Finevision and RayOne trifocal IOLs [11]. Future studies should be performed to evaluate the impact of the visual training program evaluated on eyes implanted with different types of multifocal and EDOF IOLs. Another controversial issue was the control of the distance of visualisation during the training, although subjects were trained to keep a constant distance of 40 cm during the training. Possibly, the addition of an eye-tracking system is a future advance that should be implemented, allowing monitoring the real control of such distance. Furthermore, it should be considered that the impact of this visual training has been investigated in a very homogeneous group, with a limited variation in terms of axial length, corneal power, refraction and/or comorbidities. This can be also considered as an additional potential limitation. Future research is needed to confirm if the results obtained in the current clinical trial are also obtained in other types of populations. Finally, although there were no significant differences in terms of treatment compliance, three cases of low compliance were found in the placebo group that may related to the lower engagement of the game used in this group, but this should be investigated further in future series.

One important point for future researches is the evaluation of patient satisfaction with the implant using a validated questionnaire before and after the visual training as this was not done in the current series. In the current series, all patients referred to be relatively satisfied with the results at the end of the training, but consistent conclusions about the improvement in patient satisfaction with training cannot be extracted. Future studies should be performed to compare trained vs. non-trained patients to consider this aspect. The only thing demonstrated is that photic phenomena decreased over time in placebo and study groups, but with more reduction in the level of bothersomeness of some symptoms, including glare and starbursts, in the study groups. This benefit combined with the improvement in contrast sensitivity may have improved patient satisfaction, but this has to be confirmed appropriately with validated tool for such purpose.

In conclusion, a 3-week visual training program based on the use of Gabor patches in the immediate postoperative period after the bilateral implantation of trifocal diffractive IOLs may be beneficial for improving the contrast sensitivity and the intermediate visual function, and facilitating the reduction in the complaints associated with some visual symptoms. All these changes are associated with neural changes, suggesting some level of acceleration of the neuroadaptation process. Future research is needed to optimise the effect of the training and to induce if possible a faster improvement of the visual function and a more clinically significant effect.
